# MBNL proteins and their target RNAs, interaction and splicing regulation

**DOI:** 10.1093/nar/gku767

**Published:** 2014-09-02

**Authors:** Patryk Konieczny, Ewa Stepniak-Konieczna, Krzysztof Sobczak

**Affiliations:** Department of Gene Expression, Institute of Molecular Biology and Biotechnology, Adam Mickiewicz University, Umultowska 89, 61–614 Poznan, Poland

## Abstract

Muscleblind-like (MBNL) proteins are key regulators of precursor and mature mRNA metabolism in mammals. Based on published and novel data, we explore models of tissue-specific MBNL interaction with RNA. We portray MBNL domains critical for RNA binding and splicing regulation, and the structure of MBNL's normal and pathogenic RNA targets, particularly in the context of myotonic dystrophy (DM), in which expanded CUG or CCUG repeat transcripts sequester several nuclear proteins including MBNLs. We also review the properties of MBNL/RNA complex, including recent data obtained from UV cross-linking and immunoprecipitation (CLIP-Seq), and discuss how this interaction shapes normal MBNL-dependent alternative splicing regulation. Finally, we review how this acquired knowledge about the pathogenic RNA structure and nature of MBNL sequestration can be translated into the design of therapeutic strategies against DM.

## INTRODUCTION

**Figure 1.**
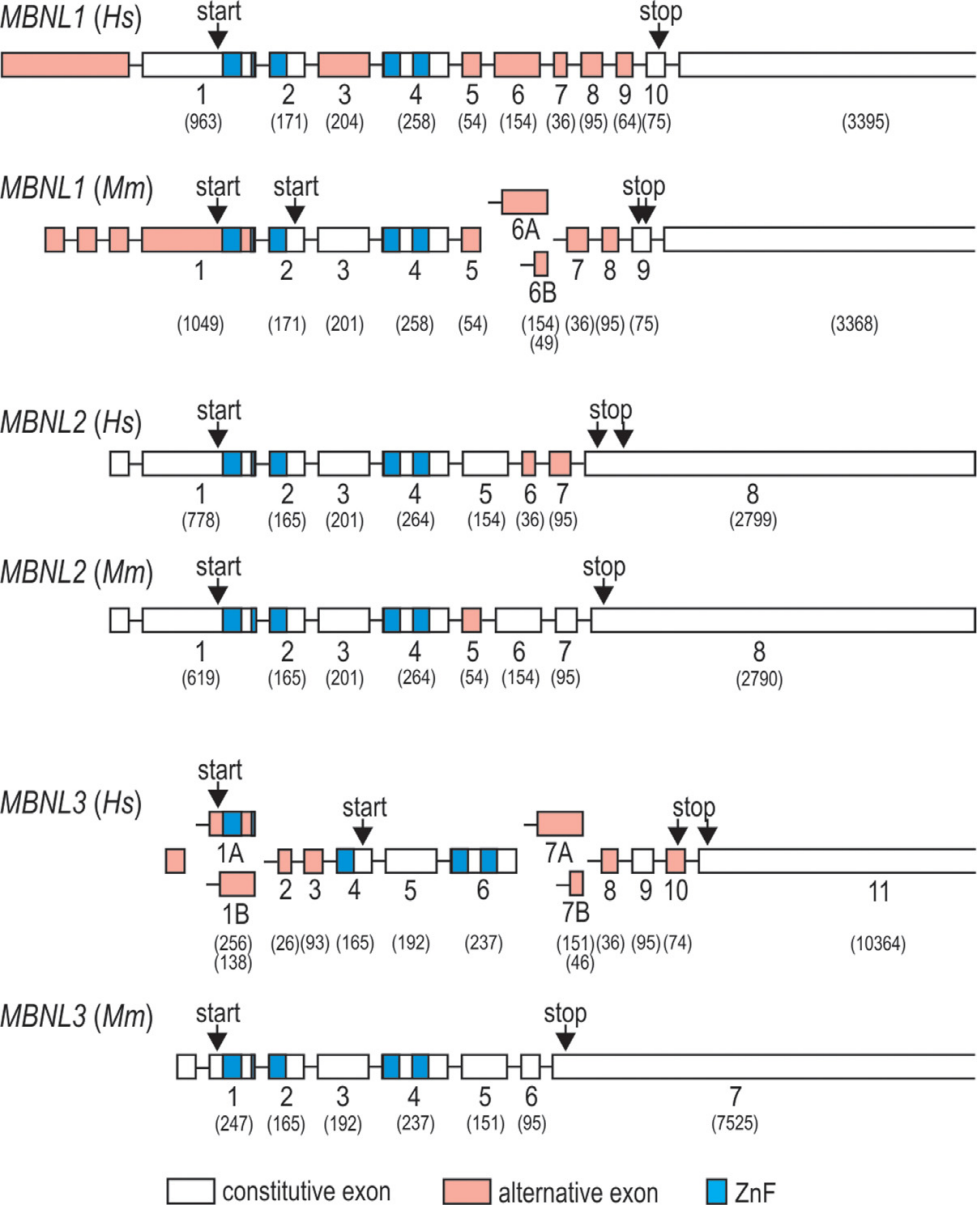
Organization of human (*Hs*) and mouse (*Mm*) *MBNL1, 2* and *3* genes. Schematic gene representations are based on sequences from RefSeq database. Exon numbers refer to protein coding exons and nucleotide length of each exon is indicated in brackets. Alternatively spliced exons and ZnFs are marked red and blue, respectively. Note that according to published data, the number of known alternative splicing isoforms of *MBNL1–3* is significantly larger than depicted in this figure. *Hs* and *Mm* indicate *Homo sapiens* and *Mus musculus*, respectively.

**Figure 2.**
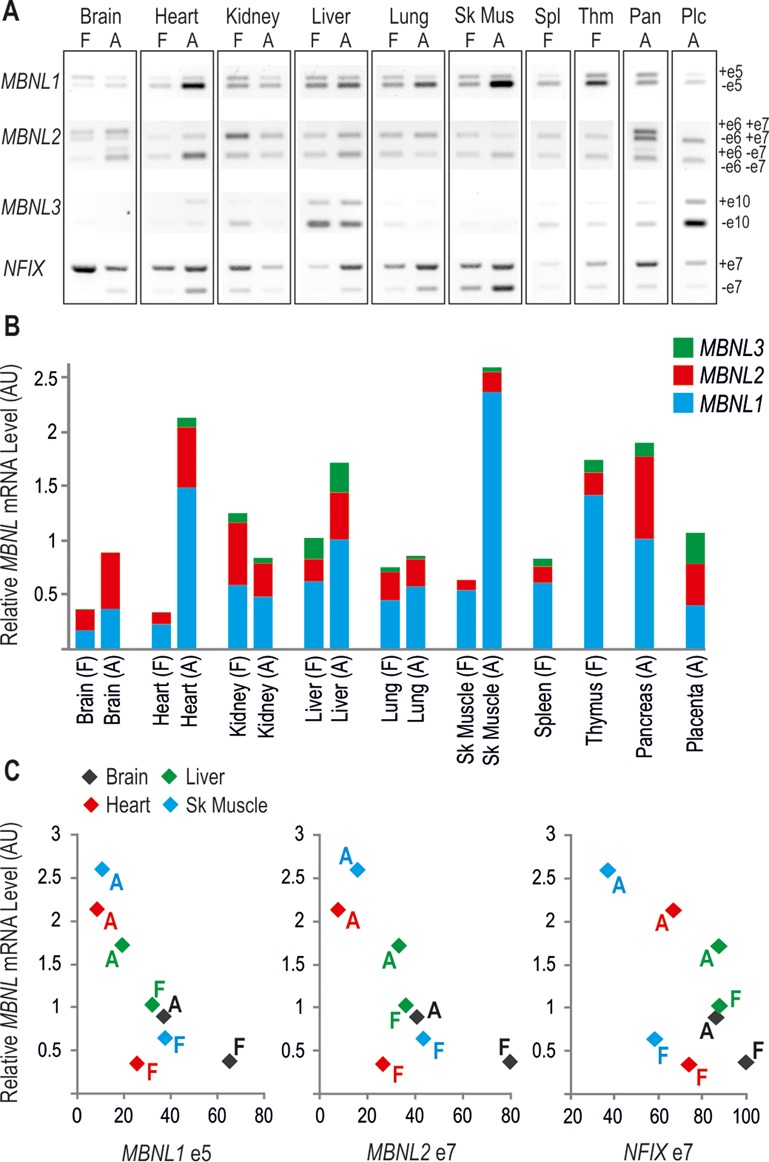
Expression pattern of *MBNL* mRNAs in different tissues and consequences on alternative splicing. Representative images of semi-quantitative reverse transcriptase-polymerase chain reaction (RT-PCR) analyses of *MBNL1* exon 5, *MBNL2* exons 6 and 7, *MBNL3* exon 10 and *NFIX* exon 7 distribution (+e, PCR band representing exon inclusion and –e, representing exon exclusion) **(A)** and multiplex PCR-based quantification of relative *MBNL1, 2* and *3* as well as total *MBNL* mRNA in various tissues **(B)**. Human fetal (F) and adult (A) cDNA panels from Clontech were used as templates for PCRs. Note the predominant *MBNL1* transcript expression in most tissues, especially in the adult skeletal muscle. Relatively high amounts of *MBNL2* are detected in the brain, and *MBNL3* in the liver and placenta. In **(C)** inclusion of *MBNL1* exon 5, *MBNL2* exon 7 and *NFIX* exon 7 was related to the total amount of *MBNL* mRNA. Note that with increasing amounts of *MBNLs* during differentiation, the splicing shifts toward MBNL-dependent exon exclusion in all depicted tissues. In contrast, splicing of *MBNL2* exon 6 does not depend on MBNL content, as exon 6 is specifically included only in the brain and pancreas [see images in **(A)**]. AU indicates arbitrary units (P. Konieczny and K. Sobczak, unpublished data).

*Muscleblind*-like proteins (MBNL) belong to a family of tissue-specific RNA metabolism regulators, which in mammals are encoded by three genes *MBNL1*, *MBNL2* and *MBNL3* [Figure 1, (1,2)]. All three family members share structural similarities, including four zinc-finger (ZnF) domains critical for recognizing a common consensus sequence in pre-mRNA and mRNA targets, but differ widely in the distribution pattern. MBNL1 and 2 are ubiquitously expressed but MBNL1 is the paralog that serves primary roles in most tissues, with the exception of the brain where MBNL2 is predominantly expressed (Figure 2B). Conversely, MBNL3 expression is much more restricted, with some functions reported in muscle cell differentiation and regeneration [Figure 2A and B, (1,3–5)]. One of the most important cellular tasks of MBNLs is tissue-specific alternative splicing regulation, in which MBNL1 and 2 have largely compensatory roles. This is indicated by a significant increase in MBNL2 expression upon functional loss of MBNL1 and profound splicing aberrations in mice lacking both MBNL1 and MBNL2 (6,7). In majority of tissues the mRNA level of *MBNL1* and *MBNL2* genes rises during differentiation. This is especially evident in the brain, heart and skeletal muscle, in which there is a several fold increase in *MBNL1* and *2* mRNA expression in adult tissues compared to the fetal (Figure 2B). Data generated in *Drosophila* indicate that myocyte enhancer factor 2 (MEF2) might be one of the transcription factors that drives the expression of *MBNL1* (8).

Increased concentrations of MBNL1 and 2 during differentiation result in at least two key developmental transitions (9–11). The first promotes differentiation of embryonic stem cells and the second induces a shift from a fetal to adult splice pattern of target mRNAs (Figure 2C). Conversely, functional down-regulation of MBNLs in humans causes adult-to-fetal alternative splicing transition (12–16). This results in myotonic dystrophy [DM, (17–21)], which besides the striated muscle, affects also other tissues, such as heart and brain. The resulting phenotype is often severely disabling and fatal due to cardiac or respiratory complications. Recent data indicate that in addition to splicing regulation, MBNLs also influence gene expression by mediating cellular mRNA transport and stability as well as microRNA (miRNA) processing (7,12,22–25).

DM is the most common muscular dystrophy in adults that affects roughly 1 in 8000 people worldwide (17,21). Loss of MBNLs activity in DM is in fact a secondary effect to the expression of pathological CUG and CCUG expansions (C/CUG^exp^) that attract and sequester in the nuclei much of the available cellular pool of MBNLs. More severe type of DM, the DM1, results from a CTG repeat expansion in the 3′UTR of the dystrophia myotonica protein kinase (*DMPK*) gene (26–28). The number of triplets reaches from 50 to several thousands and larger expansions typically correlate with more severe pathologies and an earlier onset of the disease. DM2 arises as a consequence of a CCTG multiplication in intron 1 of the CCHC-type zinc finger nucleic acid binding protein (*CNBP*) gene (29). Despite larger expansions, between 75 to more than 10 000 repeats, the disease is generally much milder, with no clear correlation between the number of the repeats and the phenotype. Although primarily considered rare, DM2 is now thought to have either comparable or even higher prevalence to DM1 in some European populations (20–21,30).

*In vitro* experiments indicate that once transcribed, expanded C/CUG repeats fold into very stable and long RNA hairpin structures, so-called toxic RNAs, that co-localize in the nucleus with MBNLs and other nuclear factors in the form of ribonuclear foci (11,31–33). This altered RNA structure, as well as its increased mass, reduce diffusion rates of mutant mRNA of *DMPK* and spliced-out intron 1 of *CNBP*, and considerably decrease the ability of mutant *DMPK* transcript to move out of the nucleus to the cytoplasm (34). Importantly, MBNLs are not just passively trapped within C/CUG^exp^ but also directly participate in foci formation, which are in fact dynamic structures (34,35). This unstable character of foci is particularly important from the therapeutic viewpoint, as it enables destabilization of interaction between MBNL and C/CUG^exp^ with small molecules. The sequestration of MBNLs directly impairs splicing of several key regulatory target pre-mRNAs in muscles and neural cells. Particularly, missplicing of muscle chloride channel (*CLCN1*), insulin receptor (*INSR*), bridging integrator 1 (*BIN1*) and calcium channel voltage-dependent L type alpha 1S subunit (*CACNA1S*) results in myotonia, insulin resistance and muscle weakness, respectively, constituting key hallmarks of DM (13,15,36–39). MBNL overexpression as well as MBNLs’ release from the C/CUG^exp^ have been shown sufficient to reverse this pathological phenotype [(40,41), see also the chapter on therapeutic strategies in this review].

In this review, we highlight the structural basis of MBNL/RNA interaction with the implication for MBNL function in differentiation and disease, and discuss how this knowledge can be shaped into potential therapeutic approaches against DM.

## MBNL DOMAINS IMPORTANT FOR RNA BINDING AND SPLICING REGULATION

MBNL1 is the best-studied family member of MBNL proteins due to its predominant expression in muscle tissue (Figure 2B). Out of twelve exons included in human *MBNL1*, ten correspond to the coding sequence (labeled here 1–10) and six (3, 5, 6, 7, 8 and 9) undergo alternative splicing (Figure 1). This results in expression of at least seven *MBNL1* mRNA variants (40,42–43). Such extensive alternative splicing regulation also occurs for other MBNLs (Figure 1), which adds to the functional range of MBNL protein family. MBNL1 variants with exons 5 and 7 occur mainly in early differentiation stages and in adult DM tissues [Figure 2A, (16,44)]. Notably, these exons enhance sequestration of MBNLs in nuclei of DM cells and thus contribute to the severity of the DM phenotype. Extensive analyses of MBNL1 deletion constructs showed that exon 5 as well as the conserved five-amino acid KRAEK motif in exon 6 compose a bipartite nuclear localization signal (43,45). Exon 7, on the other hand, determines MBNL1 dimerization as its presence induces formation of multimeric ring-like structures upon binding to toxic RNA hairpins (46) and induces MBNL1 self-association in yeast-two-hybrid assay (43).

**Figure 3.**
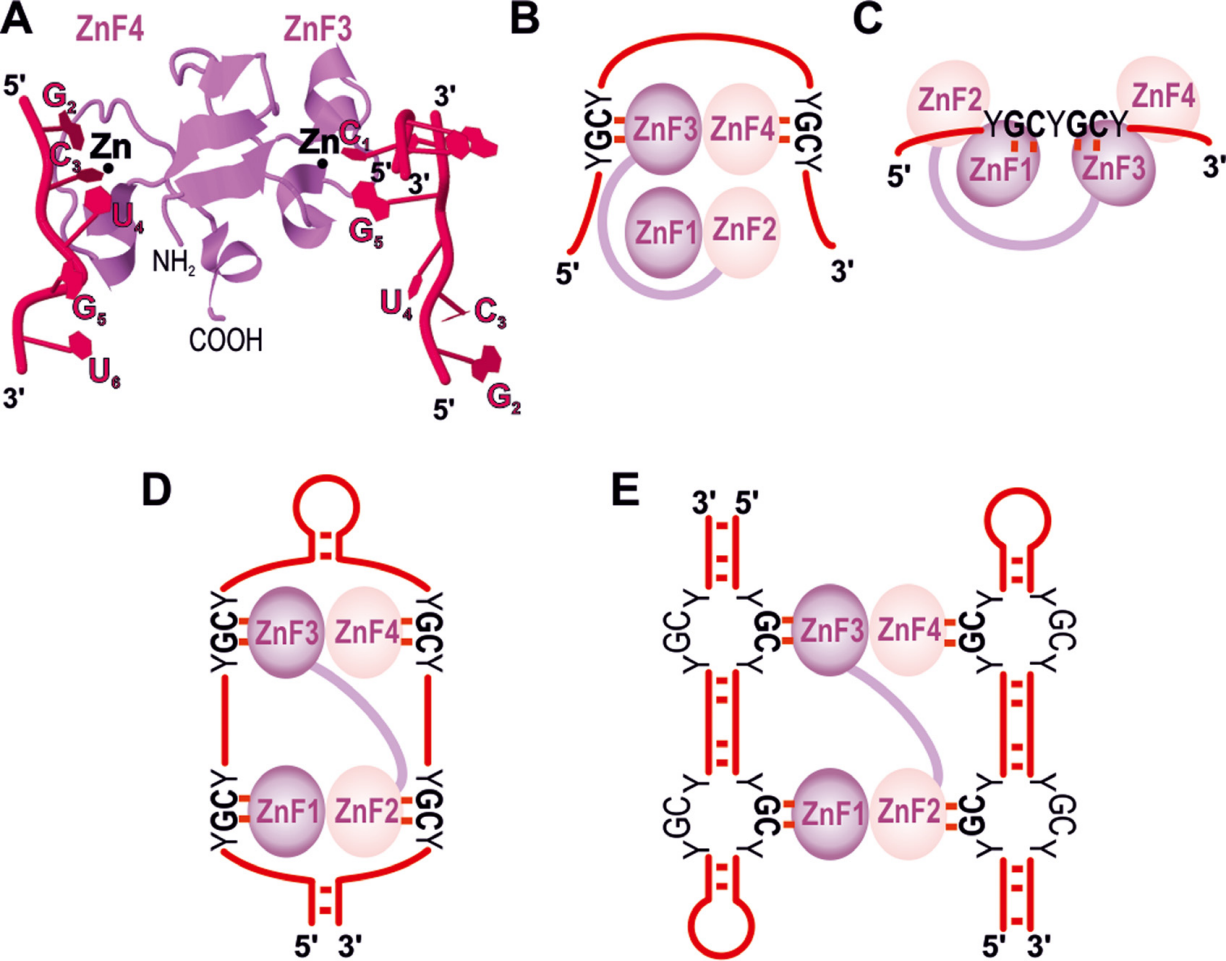
The mode of ZnFs binding to single- and double-stranded RNAs. The model shown in **(A)** is adapted from the model deposited by Teplova and Patel in RCSB Protein Data Bank [3D2S structure, (52)]. The structure shows ZnF3/4 domain interacting with three 5′-CGCUGU-3′ RNA molecules. G2, C3 and U4 residues of one RNA molecule interact with ZnF4 while C1 and G5 from two separate RNA molecules bind to ZnF3. This model indicates the GC sequence as the minimum MBNL1 RNA binding motif. Panels **(B** and **C)** and **(D** and **E)** depict hypothetical interactions of MBNL1 with single-stranded RNA and double-stranded CUG repeat hairpins with locally unwound GC motifs, respectively. (**B** and**D**) are based on (**A**) while (**C**) refers to the data obtained by Cass *et al.*, where two GC motifs are separated by only one nucleotide (51). In **(E)**, in which one MBNL1 particle bridges two CUG^exp^ hairpins, we depict a hypothetical role of MBNL1 in CUG^exp^ foci formation.

**Figure 4.**
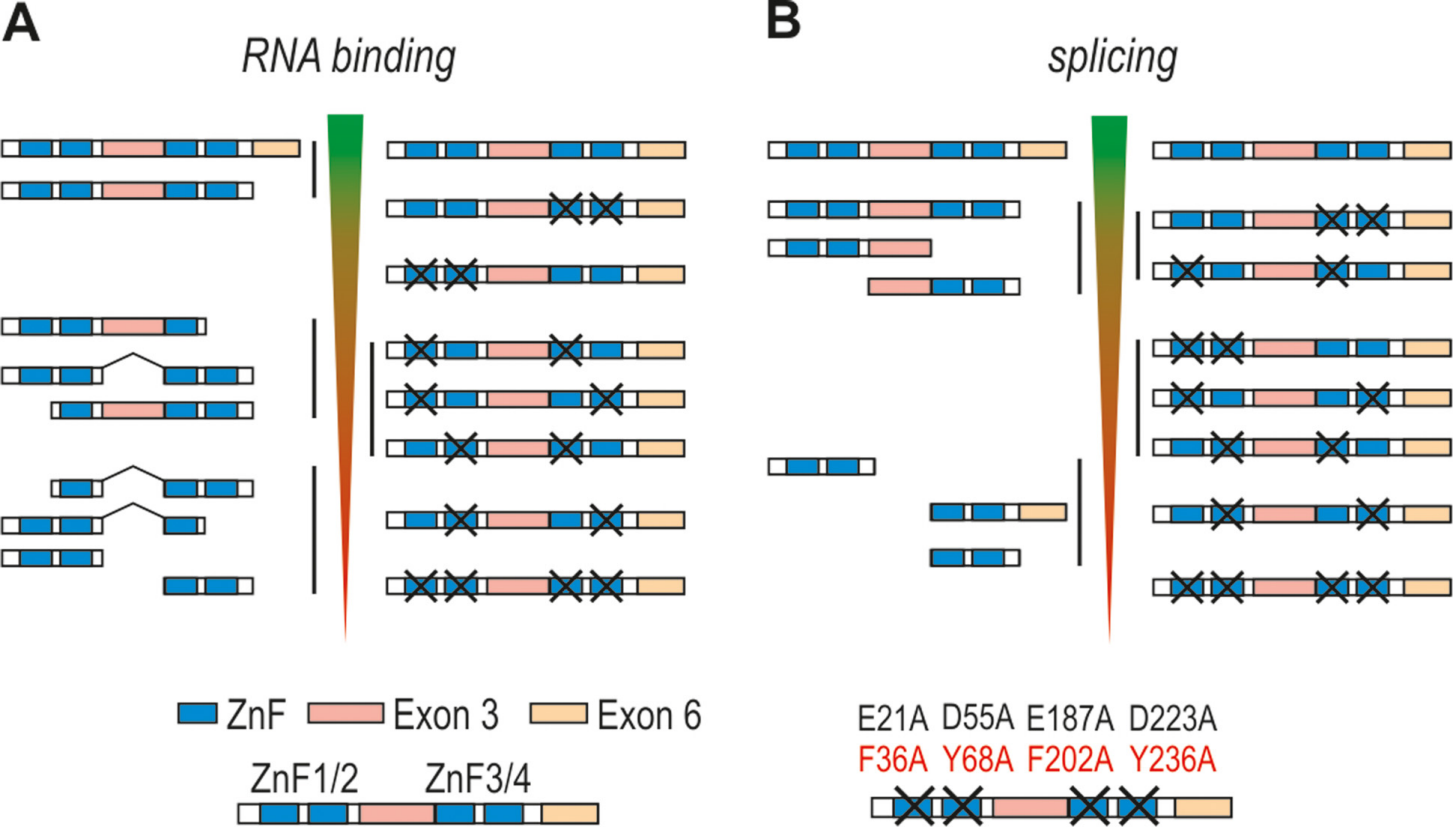
Important MBNL1 regions responsible for RNA binding and splicing regulation. Schematic representation of binding affinity of MBNL mutants to target RNAs **(A)** and their splicing activities **(B)**. Data obtained from truncation (43,47,49–50,53) and point mutation studies (47,48) are depicted on the left and right panels of **(A)** and **(B)**, respectively. In point mutation studies, key amino acids responsible for interaction with guanine and cytosine bases were substituted to alanines as marked in the figure legend. Purcell *et al.* (48) substituted two amino acids per each ZnF motif while Edge *et al.* (47) mutated only one amino acid (marked in red). Decreasing binding affinities and splicing activities of distinct MBNL1 variants are marked with inverted triangles. The top of inverted triangles indicates normal/full-length MBNL1 binding affinity and splicing activity. Protein variants showing similar RNA binding or splicing activities are grouped (black vertical lines).

Exons 1, 2 and 4 of *MBNL1* encode ZnF domains arranged in two similarly structured tandem pairs that constitute ZnFs 1 and 2 (ZnF1/2), and ZnFs 3 and 4 (ZnF3/4; Figures 1, 3 and 4). Each ZnF motif includes three cysteines followed by one histidine [CCCH, (42)]. ZnF pairs show high structural resemblance with identical spacing between cysteine and histidine residues in ZnF 1 and 3 (CX7CX6CX3H, where X is any amino acid), and ZnF 2 and 4 [CX7CX4CX3H, (1)]. Their binding affinities for target RNAs are, however, different - MBNL1 mutants with alanine substitutions of the key amino acids in the RNA binding region of ZnF1/2 have lower RNA binding affinities than mutants with corresponding substitutions in ZnF3/4 [Figure 4A, (47,48)]. Interestingly, deletion of either ZnF1 or ZnF4 alone can markedly diminish the interaction of MBNL1 with CUG^exp^ *in vivo* (49,50). The protein/RNA interaction is also determined by a linker sequence (encoded by exon 3) that separates two ZnF tandems, as its deletion severely reduces MBNL1 binding affinity [Figure 4A, (43,49)]. One possible cause for this could be that the linker supports the protein flexibility, which in turn enables MBNL1 to interact with a wide variety of targets (51). Nevertheless, MBNL1 isoforms lacking exon 3 do interact with ribonuclear foci containing toxic CUG^exp^ as shown by forced expression of GFP-tagged MBNL1 isoforms (43) and more recently, using acceptor photobleaching Förster resonance energy transfer assay (50).

Each ZnF motif may recognize a 5′-GC-3′ step in YGCY sequence elements [GpC dinucleotide is underlined, Y corresponds to pyrimidines (12,52,54)]. Crystallographic studies showed that three beta sheet linkers position the motifs in an antiparallel orientation on opposing sides of the ZnF3/4 domain [Figure 3A, (52)]. This suggests a looped conformation of the bound RNA molecule, with ZnFs domains lying between the complementary sequences (Figure 3B). Watson–Crick faces of the interacting GC dinucleotides are buried into neighboring pockets composed of conserved aromatic, arginine and lysine side chains, with two zinc-coordinated cysteines lining their bottom (52). The MBNL1/RNA interaction is based on stacking of aromatic and arginine residues with guanine and cytosine bases and a network of hydrogen bonds. Models shown in Figure 3B and D predict MBNL intercalation between complementary strands and subsequent stabilization of the secondary RNA hairpin structure. This scenario is in agreement with a reduced affinity of ZnF3/4 to RNA molecules with a short five-nucleotide spacer between two YGCY elements (52), which accordingly would incapacitate MBNL1 interaction with RNA. Work by Fu *et al.* also supports such model, as MBNL1 recognized a short double-stranded cardiac troponin T (*cTNT*) pre-mRNA fragment with a conjugated fluorophore and a quencher at the 5′ and 3′ termini and led to separation of the molecule ends, as judged by the increased fluorescence level (55). However, in this case MBNL1 binding to the outside of the molecule, as shown in Figure 3E, would bury the GC nucleotides into the protein, which could also lead to local unfolding of the RNA structure. Interestingly, MBNL1 containing all four ZnFs was able to bind two GC sequences separated by only one nucleotide, showing that the polypeptide linker between the two ZnF tandems enables MBNL1 to adopt multiple conformations and recognize a variety of RNA targets [Figure 3C, (51)]. Similar conclusions could be drawn from the study by Lambert *et al.* (56), in which MBNL1 bound to sequences with GC motifs separated by variable spacing. A recent study revealed that one MBNL1 particle binds one (CUG)_4_ molecule while four MBNL1 particles interact with one (CUG)_12_ molecule (57). Noteworthy, in this particular study researchers used only the N-terminal part of the protein containing all four ZnFs but lacking exon 7, which, as mentioned earlier, determines MBNL1 oligomerization.

MBNL1 binding affinity to its recognition sequences within target pre-mRNAs typically correlates with its splicing activity [Figure 4, (48)]. However, in addition to ZnF motifs and the linker (exon 3), a constitutive region encoded by exon 6 that does not participate in RNA binding was shown to facilitate MBNL1 activity [compare left panels of Figure 4A and B, (43,50,53)]. This raises a possibility that exon 6 determines interactions with other proteins, such as spliceosome components or other splicing regulatory factors. Noteworthy, either ZnF domains or the linker could also serve this function. MBNL truncation studies provided data indicating that the predominant splicing functions of MBNL1 are determined by exon 3 and 6 [Figure 4B, (43,53)]. In particular, Grammatikis *et al.* narrowed down regulatory regions in MBNL3 required for splicing activation of *cTNT* exon 5 and repression of *INSR* exon 11 to the linker sequence (53). In MBNL1, the linker sequence was required for splicing activation, while a small N-terminal portion of the linker, C-terminal part of exon 4 and exon 6 were necessary for effective repression. More recently, researchers tested the significance of ZnF domains in splicing activity by introducing single point mutations that disrupt MBNL1/RNA binding without affecting the overall structure of the protein [right panel of Figure 4B, (47,48)]. Furthermore, Edge *et al.* used MS2 tethering assay to test splicing activity of ZnF regions as well as exon 3 and exon 6 sequences independently of ZnF RNA binding function (47). These studies revealed that ZnF1/2 domain is predominantly required for an effective splicing of the majority of target transcripts including tropomyosin 1 alpha (*TPM1*), *INSR*, *MBNL1*, nuclear factor I/X (*NFIX*) and very low density lipoprotein receptor (*VLDLR*). Only in a subset of targets, such as sarcoplasmic/endoplasmic reticulum calcium ATPase 1 (*ATP2A1*) and *cTNT*, ZnF3/4 sufficed for high MBNL1 activity (48). Importantly, MS2 tethering assay showed that exons 3 and 6 alone are unable to facilitate MBNL1 splicing, although the linker sequence was required for optimal activity of ZnF motifs.

## STRUCTURE OF PRE-mRNA AND TOXIC RNA TARGETS

**Figure 5.**
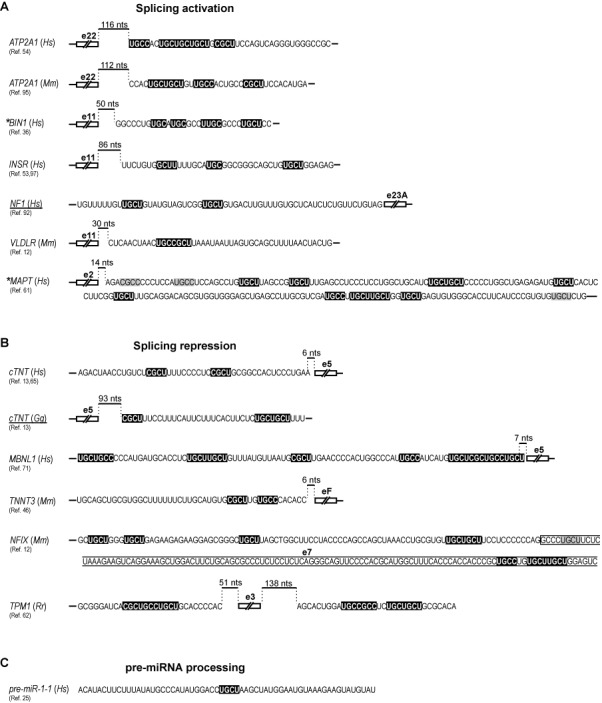
MBNL binding sequence motifs relative to the regulated exon. Exons are either positively **(A)** or negatively **(B)** regulated by MBNL depending on its position relative to the alternative exon (compare with Figure 6A). Two MBNL targets do not follow this positional pattern (underlined). Motifs in white on black background are potential MBNL interaction sites and their mutation reduce or eliminate the effect of MBNL1-dependent regulation. Note that most of them contain YGCY. Motifs in black on gray background are also potential MBNL binding sites, however, mutation of these sites either does not significantly affect MBNL1-dependent regulation [(*MAPT*), (61)] or was not tested [(*NFIX*), (12)]. Asterisks show pre-mRNA targets for which additional MBNL binding sites have been indicated. In *TPM1*, the upstream sequence is critical for exon skipping (62). In **(C)**, the MBNL1 binding motif in pre-miRNA-1–1 is indicated. Note that this is the only target containing a single MBNL consensus sequence. *Hs*, *Mm*, *Gg* and *Rr* indicate *Homo sapiens*, *Mus musculus*, *Gallus gallus* and *Rattus rattus*, respectively.

Several consequential studies identified YGCY as the consensus MBNL1 binding motif in RNA targets. Ho *et al.* identified YGCU(U/G)Y sequence in intronic regions adjacent to exon 5 of chicken and human pre-mRNA of *cTNT* [Figure 5B, (13)], the same motif sequence that was subsequently used for the described earlier crystallographic studies (52). The enrichment analysis of short RNA MBNL1-specific sequences and computational analysis based on splicing-sensitive microarray assays indicated YGCY, and particularly UGCU, as the most common MBNL1 binding motif, especially when repeated several times over a short sequence region [Figure 5, (12,54)]. Cross-link-induced mutation site analysis of data from deep sequencing of cDNA libraries obtained from ultraviolet cross-linking and immunoprecipitation [CLIP-Seq, (58,59)] of MBNL/RNA complexes confirmed that UGCU is one of the core elements of interaction not only for MBNL1 but also MBNL2 and 3 (4,7,23,60). In particular, Wang *et al.* reported enrichment of tetra-nucleotide sequences CGCU, UUGC, CUGC, UGCU and GCUU in CLIP-seq predicted binding sites (7) and Lambert *et al.* identified GCUUGCU as the most enriched heptamer in an *in vitro* screen that incorporated MBNL1 binding to a randomized pool of RNA sequences followed by deep sequencing [RNA-Bind-n-Seq, (56)]. MBNLs bind the same YGCY consensus motif in introns and 3′UTRs, indicating the same mode of interaction with various RNA structural segments and in different cellular compartments (4,7). Binding to exons and introns correlates with MBNLs’ interactions with pre-mRNA and splicing functions, whereas binding to 3′UTRs is linked to interactions with mature mRNAs and plays crucial roles in mRNA stability and cellular localization, including targeting of transcripts for localized translation and protein secretion (4,7,23–24). MBNL1 and 2 tend to preferentially interact with long 3′ UTR isoforms and thus may play a role in subcompartmentalization of isoform-specific mRNAs (7).

Experimental data suggest that MBNL proteins bind to diverse RNA structures that preferably contain unpaired U bases within the YGCY motif. A comparison of six potential MBNL1 binding sites in distinct pre-mRNAs, for example, resulted in a mixture of weak and strong stem loops with no clear correlation between the binding affinity and the target structure (54). Likewise, although MBNL1 showed a strong preference toward unpaired U bases, it tolerated either paired or unpaired GC sequences in UGCUGC, UGCUU, GCUUGC, CGCUU and GCUGCU motifs, in contrast to two other RNA-binding proteins (RBPs), CUGBP elav-like family member 1 (CELF1) and forkhead box 2 (FOX2), that favored fully single-stranded RNA motifs (56). Computational RNA folding analyses of intronic regions adjacent to alternative exons spliced across four mammalian species (ancient alternative exons) also revealed lower base pairing of U bases in MBNL1 binding motifs, such as GCUU (56). Similar conclusions could be drawn from studies on MBNL1 binding to expanded repeat sequences. Yeast three-hybrid system studies revealed that MBNL1 preferably interacts with structures containing multiple symmetric H/H or HH/HH bulges of CHHG or CHG repeat hairpin (where H corresponds to A, C or U) over repeat RNA with no bulges, such as for example, (CUG)n/(CAG)n-repeat RNA (49). Other experiments showed that in addition to CUG repeats, MBNL1 interacts with CAG and CCG repeats [Table 1, (35,46,49,63–65)], which also belong to a thermodynamically stable group of hairpin structures formed by triplet repeats (66). The stem portion of the CUG and CAG hairpin forms an overall A-helix despite periodically occurring U-U and A-A mismatched pairs (67,68). These mismatches are essential for MBNL1 recognition, as their replacement with Watson–Crick base pairs abolishes the protein binding (65). This is presumably due to weak pairing of U-U and A-A bases, which enables separation of both strands of the RNA duplex by MBNL1 and subsequent interaction of ZnFs with GC steps (52,65). Mismatched bases are commonly described as ‘wobble’ pairs with a single hydrogen bond (67,69), although the U-U pairs can adopt other conformations, depending on their position in the helix (70). For comparison, CGG triplets, also forming stable hairpins, adopt a strong two hydrogen bond between G-G pairs which seem to completely prevent MBNL1 binding [Table 1, (69)].

**Table 1.** Thermodynamic stabilities of distinct RNA structures composed of repeated sequences

Transcript	MBNL1 binding		−ΔG°_37_	*T*_m_	Reference
	*In vivo*	*In vitro*	(kcal/mol)	(°C)	
(CCUG)_n_	++++	+++		41–42	(49,65)
(CCG)_n_	++	++	∼ 2,6	59	(49,64–65)
(CUG)_n_	++/+++	++	∼ 4,6	58–63	(11,35,46,49,63–65)
(CAG)_n_	++	+	∼ 6,2	66	(11,35,46,49,63–65)
(CGG)_n_	−	−	∼ 6,7	75	(49,64–65)

*In vivo –* fluorescence *in situ* hybridization (35,63), fluorescence recovery after photobleaching (35), yeast three-hybrid system (49).

*In vitro* – photocrosslinking assays (11,46), filter binding assays (46,63), gel shift assays (46,49).

ΔG°_37_ – change in free energy at 37°C.

*T*_m_ – melting temperature.

Several studies indicate that upon binding, MBNL1 influences RNA structure stability and shifts helical targets toward locally single-stranded conformation [Figure 3, (52,55,71)]. This could indicate that MBNL1 has reduced affinity toward more structured targets, such as toxic C/CUG^exp^ (55). However, most of the studies report similar dissociation constant values for target regions of pre-mRNA and toxic CUG^exp^ (36,46,53–54,65,72). In particular, Warf and Berglund showed that MBNL1 binds an RNA-hairpin composed of 90 CUG repeats with a comparable affinity as a molecule reduced to two pairs of CUG triplets separated by a UUCG tetraloop, or a helical portion of a stem-loop in *cTNT* mRNA (65). Affinities of a GC-rich stem-loop structure containing two YGCY sequences in troponin T3 (*TNNT3*) pre-mRNA fragment and C/CUG^exp^ are also within the same range (46,72), despite their different binding kinetics (72). These data indicate that MBNL1 binds pre-mRNA targets and toxic repeats with similar affinity, and its sequestration by toxic RNA is mainly a consequence of the length of expandable repeats. While the detailed organization of CUG and CAG triplet hairpin is known, neither the nuclear magnetic resonance nor the X-ray structure of the RNA containing CCUG repeats, whose expansion causes DM2, is accessible. The current experimental data indicate that the CCUG repeat RNA also forms hairpin structures, with the stem portion adopting an A-helix conformation (73). Thermodynamically most stable model predicts two C-G base pairs divided by C-U mismatches (73) and an alternative, CG base steps with interchanging C-C and U-U mismatches (65).

Intriguingly, MBNL1 affinity for CCUG repeats is either higher or within the same range as for CUG (36,49,65,72), and given typically larger expansions in DM2 than DM1, the milder phenotype of DM2 patients remains elusive. The severity of DM1 is presently attributed not only to the toxic effect of the mutant RNA but also to the haploinsufficiency of *DMPK* as well as the local chromatin structural alterations and the resulting influence on the adjacent genes, such as sine oculis homeobox homolog 5 [*SIX5*, (17)]. Other important factors contributing to differences in DM1 and DM2 phenotype severity are the level and tissue-specific expression of mutant RNA containing either CUG^exp^ or CCUG^exp^, and localization of repeated sequences in mature mRNA of *DMPK* or spliced out intron 1 of *CNBP*, respectively. Muscles of DM1 patients also tend to have an elevated level of CELF1 (38,74–78), presumably because of its hyperphosphorylation and stabilization (76). In contrast, the level of CELF1 in DM2 tissues seems to be more variable with some patients showing no apparent difference in CELF1 level in comparison to control samples (16,77–79). Furthermore, CUG and CCUG repeats seem to interact with a different array of proteins. In a recent study, for example, depletion of a dead box helicase 6 (DDX6) in DM1 and DM2 fibroblasts increased the content of CUG^exp^- but not CCUG^exp^- containing foci (80). Finally, expanded polyglutamine tracts expressed from antisense transcripts have been detected in cells from DM1 patients (81). This could indicate expression of a different set of potentially toxic polymeric peptides from CUG and CAG expansion transcripts (DM1), and CCUG and CAGG expansion transcripts (DM2), by repeat-associated non-ATG translation (82,83).

## MBNL-DEPENDENT SPLICING REGULATION

Comparative microarray-based gene expression analyses of muscles from *HSA*^LR^ mice, expressing 250 CUG repeats in the human skeletal actin 3′UTR (84), and *MBNL1*^ΔE3/ΔE3^ mice, deficient for MBNL1 (15), revealed 80–90% similarity in splicing perturbations, proving that MBNL1 functional decrease is the key element in DM pathogenesis (12). Out of over 200 splicing events, a similar number revealed increased exon inclusion and exon skipping, showing that MBNL1 can regulate the splicing of alternative exons either positively or negatively. CLIP-Seq analyses from various mouse tissues revealed an even higher number of MBNL1-dependent alternative events, including more than 900 predicted splicing events and 500 alternative 3′UTRs (7). Interestingly, Du *et al.* (12) showed that motifs recognized by CELF1 did not co-enrich strongly with MBNL1-dependent exons, demonstrating that splicing regulation by these factors is not as tightly coupled as previously hypothesized (85). Nevertheless, data obtained from splicing microarray analyses in the developing heart as well from other studies clearly show that MBNL1 and CELF1 antagonistically co-regulate several pre-mRNA targets including *CACNA1S*, *cTNT*, H2A histone family member Y (*H2AFY*), *INSR* and *MBNL2* (13–14,39,86–87). It is worth mentioning that some MBNL1-dependent alternative exons could potentially be controlled in cooperation with other splicing factors, such as polypyrimidine tract binding protein [PTB, (62)]. The amount of MBNL1 relative to other splicing regulators could determine the strength of exon inclusion/exclusion and explain differences in MBNL1 splicing activity in distinct tissues [Figure 2C, (88,89)].

**Figure 6.**
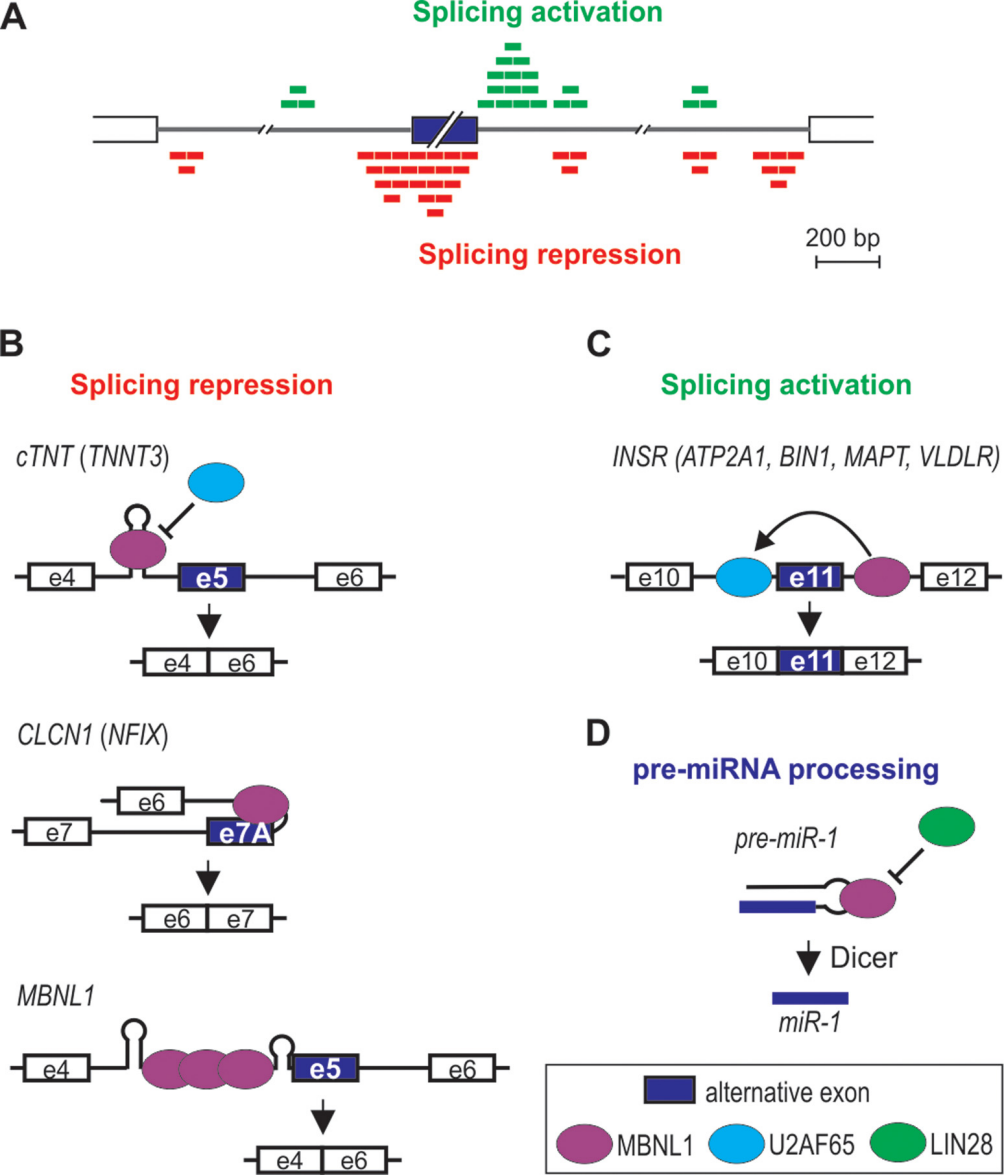
MBNL-induced alternative splicing regulation. The model depicted in **(A)** is based on data obtained from the CLIP analysis performed by Wang *et al.* (7). MBNL1 binding either upstream (red boxes) or downstream (green boxes) to the alternative exon promotes alternative exon exclusion as exemplified in **(B)** by *cTNT* exon 5, *CLCN1* exon 7A and *MBNL1* exon 5, or exon inclusion as exemplified in **(C)** by *INSR* exon 11. The mode of MBNL1 regulation of transcripts in parentheses is putative. Interaction of MBNL1 with pre-miRNA hairpin promotes pre-miRNA processing to mature miRNA **(D)**.

MBNL splicing function depends on its binding position relative to the regulated exon [Figures 5 and 6A, (7,12,54,60)]. MBNL1 and 2 binding within the alternative exon and upstream intronic regions generally facilitates exon skipping, while binding to downstream intronic regions promotes exon inclusion. This pattern of splicing regulation is akin to other factors, such as neurooncological ventral antigen (NOVA), FOX or heterogeneous nuclear ribonucleoprotein L [hnRNP L, (90)]. Such configuration indicates that repression of exon inclusion is a consequence of MBNLs directly blocking the exonic enhancers, 3′ splice-sites, polypyrimidine-rich tracts or intronic branch site elements, whereas activation might be caused by MBNLs competition with splicing silencers, activation of intronic splicing enhancers or enhanced recognition of 5′ splice-site by spliceosome (7,9,54,91). Other positional splicing activities of MBNL1 also occur [Figures 5 and 6A, (3,7,10,13,23,92)]. Of these, exon exclusion by binding to the 3′ splice-site in the downstream intronic region appears to be the most common (7,23). Also, in embryonic stem cells where MBNL1 level is very low, high-throughput RNA sequencing combined with a splicing code analysis revealed a reversed regulatory pattern to the one depicted in Figure 6A (10).

As already mentioned, MBNL can interact with a variety of different RNA structures containing YGCY sequence motifs. One mode of MBNL action might be via binding and stabilization of a stem loop structure (Figure 6B). For example, negative regulation of *CLCN1* exon 7A occurs by MBNL1 binding to a hairpin in the junctional region between intron 6 and short exon 7A, which occludes the polypyrimidine tract, 3′ splice site and the exonic-splicing enhancer (37). MBNL1 can also recognize an intronic RNA stem-loop structure adjacent to the 3′ splice-sites of a negatively regulated exon F of *TNNT3* (15,40,46). Similarly, the data by Warf *et al.* indicate that MBNL1 interacts with a stem-loop structure in a portion of the intron at the 5′-end of an alternatively regulated *cTNT* exon 5, which blocks the splicing factor U2AF65 from recognizing the polypyrimidine tract (93). This in turn inhibits U2 snRNP recruitment that facilitates exon 5 skipping. RNA helicase p68 (DDX5) enhances MBNL1 binding to the stem-loop as well as toxic CUG^exp^ (94). As a possible scenario, Laurent *et al.* suggested that mismatches in the RNA structure provide an anchoring site for the helicase to initiate the strand separation, facilitating the interaction of MBNL1 with consensus binding motifs. Interestingly, another dead box helicase DDX6 was recently found to exert an opposite effect (80). Upon overexpression, DDX6 induced relocalization of CUG^exp^ to the cytoplasm, dissociation of MBNL1 from mutant transcripts and partial correction of splicing aberrations.

Crystallization study showed that MBNL1 ZnF3/4 binds to single-stranded CGCUGU sequences (52). Correspondingly, there is no evidence of a hairpin formation in the MBNL1 binding site of *INSR* or *ATP2A1* pre-mRNA (53,95–97). In the former, MBNL1 binds to the intronic splicing enhancer in the downstream intron of the regulated exon (Figure 6C). This activates U2AF65 interaction with the upstream intron possibly through MBNL1-induced change in RNA conformation or assembly of other proteins and a subsequent excision of the upstream intron (91). The removal of the downstream intron, on the other hand, is not dependent on MBNL1, despite the direct interaction of MBNL1 with this region. MBNL1 might also induce transition of a hairpin structure into single-stranded conformation as in intron 4 of *MBNL1* pre-mRNA (Figure 6B). Here, MBNL1 binding within AG exclusion zone downstream of a distant branchpoint and polypyrimidine tract causes RNA unwinding, allowing more MBNL1 protein molecules to interact with the complex, which in turn inhibits spliceosome from locating the 3′ splice-site (71). MBNL1 might also interact with an unpaired region of a terminal loop of hairpin structures. Rau *et al.* showed that MBNL1 binds a single UGC motif located within a loop of pre-miR-1 hairpin [Figure 6D, (25)]. MBNL1 binding prevents pre-miR-1 interaction with LIN28, which otherwise would induce pre-miR-1 uridylation by TUT4 and blockage of Dicer processing, resulting in a decrease of miR-1 expression observed in DM1 muscles. Intriguingly, pre-miR-1 is the only reported target with a single MBNL1 consensus sequence (Figure 5C).

## THERAPEUTIC STRATEGIES TARGETING PATHOGENIC MBNL/RNA INTERACTION

Currently, no cure exists for the complex pathobiology and wide-ranging clinical manifestations of DM, and patients’ therapy is limited to symptomatic treatments and supportive care. However, several distinct molecular intermediates of the disease, such as the expanded C/CUG repeat RNA and the toxic MBNL/RNA complex, may constitute potential points of therapeutic intervention (98). While majority of currently available therapeutic strategies are designed to either reduce or eliminate the expression of toxic transcripts, in this review we only highlight approaches that seek to target the interaction between C/CUG^exp^ RNA and MBNL, constituting the root-cause of DM. In particular, we focus on small chemical compounds and antisense oligonucleotide-based strategies that are specifically designed to disrupt the structure of the complex or to prevent its formation. Both strategies rely on a presumption that small molecules or antisense reagents could specifically bind the C/CUG repeat hairpin with higher affinity than MBNL, hence releasing it from pathogenic sequestration and boosting the cellular pool of available MBNL for interaction with pre- and mRNA targets.

First examples of small molecules targeting expanded CUG repeat hairpin were identified by Gareiss *et al.* in a study utilizing a resin-bound dynamic combinatorial chemistry screen (99). Recent refinement of these molecules led to compounds active *in vivo*, which were able to partially restore proper MBNL1-dependent alternative splicing in the *HSA*^LR^ mouse model (100). Similarly, a screen of library of small compounds already known to bind to structured RNA identified pentamidine, a molecule initially speculated to release MBNL1 from CUG^exp^ by binding the minor groove of the A-form CUG repeat hairpin (101). However, a follow-up study by Coonrod *et al.* proved otherwise, as it turned out that CUG^exp^ transcripts dropped significantly upon pentamidine treatment, suggesting that the molecule is not directly blocking MBNL1 binding but rather inhibiting transcription of CTG/CAG repeat DNA or increasing the rate of CUG^exp^ RNA turnover (102). Nonetheless, other studies strongly supported the idea of using small molecules capable of binding expanded CUG repeats to either release MBNL1 from toxic RNA or block its sequestration. One example is D-amino acid hexapeptide (ABP1) that not only reduced CUG^exp^ foci formation and suppressed CUG^exp^-induced lethality and muscle degeneration in DM flies, but also reversed muscle histopathology and splicing misregulation of MBNL1 targets in a DM1 mouse model (103). *In vitro* studies showed that ABP1 did not prevent MBNL1 binding to CUG hairpins, but instead induced a conformational shift in the secondary structure of CUG RNA from predominantly double-stranded hairpins to single-stranded CUG repeat molecules, which in turn blocked further MBNL1 sequestration (103). In another study, Jahromi *et al.* reported a cell penetrable small molecule that targets CUG^exp^ RNA and inhibits its interaction with MBNL1, resulting in foci dispersal and correction of *INSR* pre-mRNA alternative splicing in DM1 cell model (104). Similarly, bismanidum inhibitor reported by Wong *et al.* was able to bind toxic CUG-repeat RNA and disrupt its interaction with MBNL1 *in vitro*, and in addition dispersed CUG^exp^ foci and corrected misregulated splicing of *cTNT* and *INSR* when applied to a DM1 cell model (105).

Recently, a time-resolved fluorescence resonance energy transfer screen of the Molecular Libraries Small Molecule Repository (MLSMR), comprised of roughly 300 000 small molecules, was employed to identify chemical compounds that disrupt the MBNL1/CUG^exp^ complex either by binding the protein, or the RNA (106). One of the identified compounds, a substituted naphtyridine, was able to inhibit the complex formation by interacting with the U-U loops in expanded CUG repeat RNA and displacing the MBNL1, which resulted in a significant improvement of the alternative splicing defects in a DM1 cell culture model. Importantly, this compound appeared much more selective toward CUG repeat hairpin structural motifs than previously described compounds, for example, bis-benzimidazole Hoechst (107). However, not all small molecules able to disrupt the MBNL1/RNA complex have a therapeutic potential. The same study, for instance, identified a thiophene-containing small compound that disrupted the MBNL1/CUG^exp^ complex by binding MBNL1 within ZnF domains and precluding its interaction with expanded CUG repeat RNA (106). This interaction adversely affected alternative splicing events in normal human cell lines, leading to DM1-like splicing shifts of MBNL1-dependent exons. In addition, a recent study by Hoskins *et al.* demonstrated that some ligands for CUG^exp^ repetitive sequences, apart from disrupting the MBNL1/RNA complex, might reduce toxic RNA decay (108). This work employed a high-throughput screen of the MLSMR to search for novel inhibitors of MBNL1/(CUG)_12_ interaction and demonstrated that one of the hits, a natural antimicrobial agent lomofungin, not only inhibited this interaction *in vitro* but also dispersed nucleoplasmic localization of MBNL1, and increased its splicing regulatory activity in CUG^exp^ expressing cells (108). However, lomofungin spontaneously dimerized in dimethyl sulfoxide (DMSO) producing dilomofungin, that despite a 17-fold more potency to disrupt MBNL1/(CUG)_12_ *in vitro* compared to the monomer, caused a large increase of CUG^exp^ RNA in nuclear foci when applied to cells, owing to reduced decay of the CUG^exp^ transcripts via unknown mechanism (108). Intriguingly, earlier studies with transgenic fruit flies expressing CTG repeats in a 3′UTR of a reporter gene, demonstrated stabilization of cytoplasmic CUG repeat transgenic RNA upon overexpression of *Drosophila* Muscleblind isoforms, MblA and MblC, thus indicating Muscleblind proteins as potential players in the regulation of CUG repeat transcripts stability (109).

More recently, an alternative strategy was developed based on a rational design of modularly assembled ligands that target and neutralize CUG^exp^ hairpins. Modularly assembled ligands offer the advantage of targeting many RNA structure repeat units at a time, and hence offer more specificity toward expanded repeat RNA, higher potency and potentially, less off-target effects (i.e. binding natural mRNA targets that contain short CUG repeats). For example, derivatives of Hoechst 33258 assembled modularly into a peptoid scaffold displaying multiple copies of the ligand separated by spacing modules were highly specific in binding multiple CUG-repeat motifs *in vitro* with low nanomolar affinities (110,111). The most potent pentameric ligand demonstrated 23-fold higher affinity toward CUG-repeat RNA than MBNL1 *in vitro* (111). In addition, multivalent ligands based on Hoechst derivatives efficiently disrupted nuclear CUG^exp^ foci and rescued the *cTNT* pre-mRNA alternative splicing defect in a cellular DM1 model (112). Furthermore, based on a previously reported triaminotriazine-acridine based Ligand 1, which bound U-U mismatches within CUG repeat hairpin with high nanomolar affinity and destabilized the MBNL1/CUG^exp^ complex *in vitro* at low micromolar concentrations (113), Jahromi *et al.* developed cell- and nucleus-permeable dimeric ligand with oligoamine linkers, which very effectively dispersed ribonuclear foci in DM1 cell models (114). Noteworthy, additional studies that explored small molecule compounds structurally similar to Ligand 1 demonstrated that triaminopyrimidine-based derivatives of this ligand exhibited not only strong inhibition of the MBNL1/CCUG interaction, but also high selectivity for DM2-specific CCUG repeats over other RNA targets (115).

Shape- and chemistry alignment-based virtual computational screens can also be successfully employed to improve the efficacy of CUG^exp^-binding ligands. Two recent studies based on these approaches yielded a series of inhibitors of the toxic MBNL/RNA complex, which were much more potent than the query compounds Hoechst 33258 (107) and 4′,6-diamidino-2-phenylindole (116). Importantly, the spacing between RNA-binding modules and their flexibility seem to be crucial factors affecting the potency, RNA binding affinity and specificity of such modularly assembled compounds (117,118). In addition, an important factor determining the efficacy of small compounds is their cellular uptake by a variety of cell lines as well as their optimal subcellular localization. Rational design can be successfully applied to manipulate these parameters in order to achieve optimal bioactivity of small molecules. One good example is a modularly assembled 6′-N-5-hexyonate kanamycin A (K-ligand) displayed on a peptoid scaffold that simultaneously targets several internal loops within the DM2-specific CCUG-repeat hairpin and binds it more tightly than MBNL1 (119). Appropriate spacing between the ligand modules can strongly affect the binding selectivity of such a modularly assembled compound, as a follow-up study demonstrated that decreasing the number of spacing modules in K-ligand made it more selective toward DM1-specific CUG-repeat RNA over the DM2-specific CCUG-repeat RNA (120). However, even though it showed efficient cellular uptake and low levels of toxicity (120), it was not appreciably active in cellular models of DM1 due to suboptimal cellular permeability and localization (121). Interestingly, by conjugating it to a cellular uptake tag, a D-Arg_9_ molecular transporter, researchers were able to improve the ligand's cellular uptake and bioactivity and consequently improve DM1-associated defects in cell and animal models (121).

The neutralization of RNA toxicity can be also achieved by antisense oligomers targeting CUG repeats. However, majority of available strategies that fall into this category are based on degradation of CUG^exp^ RNA. Some of the most recent examples include the use of gapmers to promote target cleavage via RNaseH (122), or the use of small interfering RNAs (siRNA) to induce CUG^exp^ degradation via RNA interference pathway (123), as well as application of antisense oligomers with variously modified nucleotide chemistries to silence the expression of mutant transcripts (124–126). The only antisense oligomer described so far that efficiently binds CUG repeat RNA and precludes MBNL1 sequestration without inducing significant degradation of the toxic transcript is a 25-mer morpholino CAG25 reported by Wheeler *et al.* (41). This study demonstrated that local injection of CAG25 into skeletal muscles of *HSA*^LR^ mice decreased the number of CUG^exp^ foci, redistributed MBNL1 protein, corrected abnormal alternative splicing of MBNL1-sensitive exons and reduced myotonia (41). It also led to a slight reduction in the steady-state levels of the *HSA*^LR^ transgene mRNA, which the authors attributed to enhanced natural decay of the transgene transcript due to its relocation to cytoplasm, as CAG25 was unable to support RNA cleavage by RNaseH. Importantly, the authors also postulated that the therapeutic effect of CAG25 likely results from displacement of the MBNL1 protein from the expanded CUG repeats (41). Furthermore, Leger *et al.* utilized CAG25 with a 5′ primary amine modifications, linked to arginine-rich cell penetrating peptide, for a systemic administration into *HSA*^LR^ mice to demonstrate that it efficiently redistributes MBNL1 protein in myonuclei and corrects alternative splicing defects (127).

Summarizing, expanding knowledge of the structural basis for MBNL/RNA interaction opened up several options for researchers to prevent the pathogenic protein/RNA complex formation or revert its toxic effect. The future challenge lies in achieving effective cellular delivery and ensuring optimal bioactivity of the therapeutic compounds. Finally, in the case of small molecules targeting the RNA component of the DM complex, increasing their specificity toward expanded CUG repeat RNA and minimizing the off-target effect toward other CUG repeat-containing RNAs will be yet another demanding task in a therapeutic battle against DM.

## FUTURE PERSPECTIVES

In this review we highlighted the structural basis for MBNL1 target recognition and discussed how it impacts the regulation of alternative pre-mRNA splicing in differentiation and disease. MBNLs bind the common YGCY motif in pre-mRNA and mRNA. However, other factors, such as proximity of additional YGCY motives, pyrimidine content of adjacent regions and RNA structure, might also play a role in MBNLs’ target recognition (54–56). It is still elusive to what extent each of these factors shapes the MBNL/RNA interaction. With the dawn of CLIP and deep RNA sequencing, more detailed splicing maps of various RBPs are generated. Despite binding to different motives, many of them share common positional principles, presumably because of a similar effect they exert on spliceosome or serine/arginine-rich components. This is especially apparent in alternative exon exclusion where RBPs often occlude spliceosome interaction sites (90). Of interest is creation of alternative splicing tissue-specific maps. It is becoming apparent that majority of alternative splice events are tissue-specific and that many of them could be controlled by heterotypic RBP interactions (90,128–129). It is still unclear how MBNL-dependent targets are regulated in relation to other RBPs. CELF1 antagonistically controls some MBNL targets, however, the extent to which both RBPs co-regulate alternative splice events seem to differ between various tissues (12,14). Another recently emerging aspect is MBNL's interaction with other proteins, not only in the context of splicing regulation (91), but also during the transport of mature mRNAs to various cellular compartments, some of which might involve carrying RNA molecules along the various cytoskeletal elements (7). Delineation of the intricate molecular interactions of MBNLs will help design more successful therapies against DM in the future.
